# Novel group-based QSAR and combinatorial design of CK-1δ inhibitors as neuroprotective agents

**DOI:** 10.1186/s12859-016-1379-9

**Published:** 2016-12-22

**Authors:** Kopal Joshi, Sukriti Goyal, Sonam Grover, Salma Jamal, Aditi Singh, Pawan Dhar, Abhinav Grover

**Affiliations:** 1 Amity School of Biotechnology, Amity University, Noida, Uttar Pradesh 201303 India; 2 Department of Bioscience and Biotechnology, Banasthali University, Tonk, Rajasthan 304022 India; 3 Kusuma School of Biological Sciences, Indian Institute of Technology Delhi, New Delhi, 110016 India; 4 Department of Biotechnology, TERI University, New Delhi, 110070 India; 5 School of Biotechnology, Jawaharlal Nehru University, New Delhi, 110067 India

**Keywords:** Amyotrophic lateral sclerosis, ALS, TDP-43, Casein kinase, QSAR, Combinatorial library

## Abstract

**Background:**

Tar DNA binding protein 43 (TDP-43) hyperphosphorylation, caused by Casein kinase 1 (CK-1) protein isoforms, is associated with the onset and progression of Amyotrophic Lateral Sclerosis (ALS). Among the reported isoforms and splice variants of CK-1 protein superfamily, CK-1δ is known to phosphorylate different serine and threonine sites on TDP-43 protein in vitro and thus qualifies as a potential target for ALS treatment.

**Results:**

The developed GQSAR (group based quantitative structure activity relationship) model displayed satisfactory statistical parameters for the dataset of experimentally reported N-Benzothiazolyl-2-Phenyl Acetamide derivatives. A combinatorial library of molecules was also generated and the activities were predicted using the statistically sound GQSAR model. Compounds with higher predicted inhibitory activity were screened against CK-1δ that resulted in to the potential novel leads for CK-1δ inhibition.

**Conclusions:**

In this study, a robust fragment based QSAR model was developed on a congeneric set of experimentally reported molecules and using combinatorial library approach, a series of molecules were generated from which we report two top scoring, CK-1δ inhibitors i.e., CHC (6-benzyl-2-cyclopropyl-4-{[(4-cyclopropyl-6-ethyl-1,3-benzothiazol-2-yl)carbamoyl]methyl}j-3-fluorophenyl hydrogen carbonate) and DHC (6-benzyl-4-{[(4-cyclopropyl-6-ethyl-1,3-benzothiazol-2-yl)carbamoyl]methyl}-2-(decahydronaphthalen-1-yl)-3-hydroxyphenyl hydrogen carbonate) with binding energy of −6.11 and −6.01 kcal/mol, respectively.

## Background

Amyotrophic Lateral Sclerosis (ALS) is a progressive neurodegenerative disease which results in paralysis, muscle wasting and death. Death of the motor neurons of the cortex, spinal cord and brain stem is a characteristic of this disease which eventually leads to death of the patient usually resulting from respiratory failure, mostly within 3–5 years from the appearance of symptoms [1]. The term “Amyotrophic” refers to muscle atrophy and weakness which are characteristic to the lower motor neuron disease while “Lateral Sclerosis” occurs due to hardness to palpitation of the lateral columns of spinal cord observed in autopsy specimens [2]. The prevalence or occurrence of the disease is about 3 per 100,000. The ratio of male:female prevalence of ALS is 1.8-2.0:1 [3]. ALS is usually classified as familial (fALS) and sporadic (sALS) where fALS is usually inherited as a dominant trait and is observed in approximately 10% of the total cases of ALS while sALS occurs in people who do not have any apparent history of this disease in their families [4–6].

The first symptoms of ALS include twitching of muscle, stiffness, cramping and weakness later followed by difficulty in chewing, swallowing, slurred speech and difficulty in fast eye movements [7]. Also, there is difficulty in breathing as there is weakening of muscles of respiratory system. Many deaths are caused by respiratory failure within 2–10 years of onset of disease or are caused due to pneumonia [8, 9]. ALS can be caused by mutations in many different genes such as SOD1 (superoxide dismutase1), TARDBP (transactive response DNA binding protein), and C9orf72 amongst many others [10]. Recently, pathological TDP-43 (transactive response DNA binding protein 43kDA) protein, encoded by TARDBP, has been identified in sALS, which gives scope for development of therapeutic agents [1]. The protein binds to both DNA and RNA. By binding to the former, it regulates the process of transcription and is also involved in the splicing and maturation of mRNAs [11]. Neuronal death is known to be caused in mice, zebrafish, worms, flies and monkeys due to the over expression of mutant TDP-43. It is believed the phosphorylation of TDP-43 may cause toxicity of the protein [1]. Casein Kinase-1 (CK-1) was the first kinase that has been reported to cause this pathological phosphorylation of the TDP-43 protein and its activity has also been found upregulated in spinal cord tissue in ALS and other neural disorders [12]. CK-1 is a unique family of serine/threonine protein kinases that express ubiquitously in the eukaryotes. Seven CK-1 isoforms, namely *α*, β, γ1, γ2, γ3, δ and ɛ and various splice variants have been reported in mammals [13, 14]. CK-1 family member proteins have a very high (53–98%) identity in the kinase domain and differ from other kinases by containing S-I-N sequence instead of an A-P-E in kinase domain number VIII [15]. CK-1 isoforms are involved in regulation of circadian rhythms, Wnt signaling, nucleo-cytoplasmic shuttling of transcription factors, DNA transcription and DNA repair [16, 17]. They have been found upregulated and mutated in various forms of cancer [18] and neurodegenative diseases. Among the known CK-1 variants CK-1δ has been found upregulated in various neurodegenrative diseases and known to phosphorylate TDP-43 at various sites [19], so in recent years, its importance as a leading target has been highlighted through various studies. CK-1δ inhibition was found be beneficial in cancer inducing the DNA damage and also significant role in pathological TDP-43 phosphorylation have been disclosed [20, 21].

Finding brain penetrant inhibitors of CK-1δ could prevent the occurrence of this phenomenon and present a strategy for the effective treatment of ALS.

Ligand based drug designing is one of the in silico based methods which aides in establishing a quantitative relationship between the structures of inhibitors and their inhibitory activities. The quantitative structure and activity relationship (QSAR) approach attempts at identifying and quantifying the relationship between molecular structures and certain physico-chemical structures thereby producing a model for the prediction of the data [22]. The QSAR approach is also described as one in which data analysis methods are applied to develop models for accurate prediction of biological activities of molecules on the basis of their structures [23]. A regression equation is obtained which explains the variation of one or more dependent variables (biological activity) in terms of independent variables (descriptors) [24]. This study makes use of fragment-based GQSAR modeling to correlate the biological activity of the N-Benzothiazolyl-2-Phenyl Acetamide derivatives with certain physico-chemical descriptors. These molecules have been reported to prevent TDP-43 phosphorylation in the in-vitro studies and have the ability to cross the blood-brain barrier. Group-based QSAR or GQSAR is a method which investigates the structure activity relationship based on molecular fragments of the set of molecules [25–32]. GQSAR is an advantageous and more informative approach than other conventional 2D and 3D QSAR methods. Conventional QSAR methods do not make clear exactly which part of the molecules should be substituted or modified in order to increase the activity. Unlike conventional QSAR methods, GQSAR is a recent fragment based approach that provide useful information about the significant substitution sites, their chemical nature as well as overall interaction that effects the activity of molecules [33–35]. The GQSAR model instead of analysing whole molecule, evaluates molecular fragments. The biological activity of molecular fragments and their descriptors are correlated, leading to QSAR model(s) which focuses on important substitution site with their chemical nature and interactions. The information derived from the developed model helps in suggesting significant molecular fragments that can be utilized as the building blocks while designing novel molecules [36].

The focus of this study was to perform fragment based QSAR modeling on a congeneric set of N-Benzothiazolyl-2-Phenyl Acetamide derivatives. This congeneric set of compounds has been developed by Salado et al. [1]. They have developed 55 molecules as N-Benzothiazolyl-2-Phenyl Acetamide derivatives by substituting chemical moieties and changing the linker that shows a increase or decrease in the molecule's inhibitory activity. There are more molecules showing inhibitory effect against CK-1δ but we have taken 37 molecules as these show greater than 60% inhibitory activity as well as have been generated by replacement at similar number of sites i.e., 6 while other compounds have different linker chain and replacement at 3 sites only. In this study, a GQSAR model based on N-Benzothiazolyl-2-Phenyl Acetamide derivatives was built. The model helps in the explanation of the variation of the biological activity of these derivatives as a function of their site-specific molecular fragments. The GQSAR model presented certain important descriptors which were essential for the compounds to exhibit an enhanced inhibitory activity. In this study, using combinatorial library approach we report novel lead compounds with inhibitory properties against CK-1δ [37, 38]. Through this study, it has been attempted to understand how different substituents at the different positions in the representative template structure of the ligands affects its inhibitory properties, in addition to predicting the biological activities of the designed lead compounds generated in the combinatorial library.

## Methods

### Preparation of the dataset

**Fig. 1:**
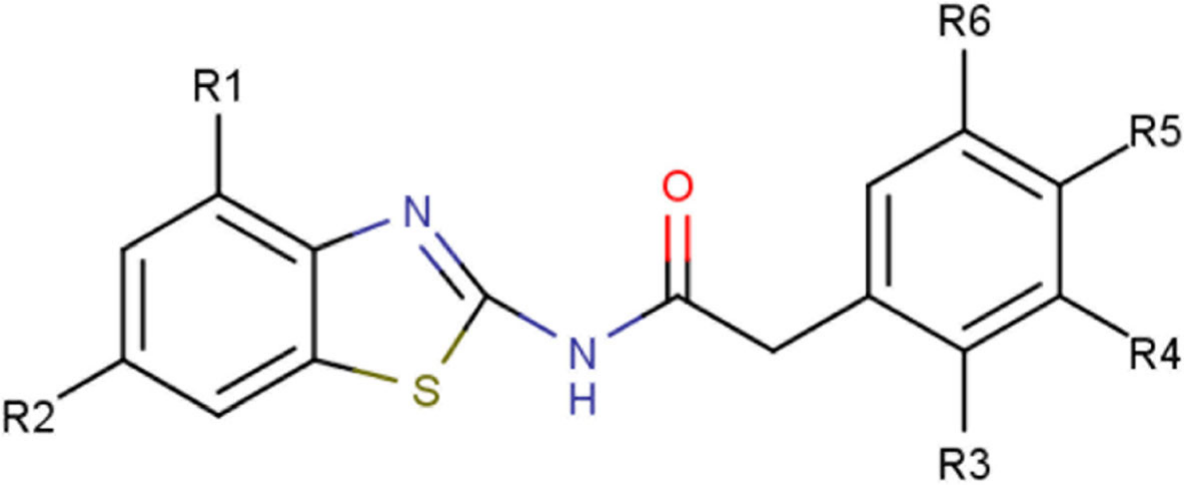
Structure of template of N-Benzothiazolyl-2-Phenyl Acetamide derived compounds. (Heteroatoms are shown in different colors; as Nitrogen in *blue*, oxygen in *red* and sulfur in *green*) & (R1, R2, R3, R4, R5 and R6 are potential substitution sites)

The structures of the congeneric dataset of 37 N-Benzothiazolyl-2-Phenyl Acetamide derivatives was prepared using MarvinSketch [1, 39]. The 2D structures were converted into 3D structures using the VLifeEngine module of VLifeMDS [40]. Energy minimization of 3D compounds was performed with the help of force field batch minimization module of VLifeEngine. This step is performed to optimize the molecules upto their lowest stable states of energy. The template was also drawn using MarvinSketch, keeping a common structural moiety in congeneric dataset of all N-Benzothiazolyl-2-Phenyl Acetamide derivatives. The template has 6 substitution sites, marked by dummy atoms and depicted as R1–R6 (Fig. 1).

### Calculation of descriptors for GQSAR modeling

This step is performed using the GQSAR module of VLifeMDS [40, 41]. The pIC50 values of the compounds were incorporated into VLifeMDS manually which was followed by the calculation of various 2-D physico-chemical descriptors for the different functional groups present at different substitution sites of the compounds (Refer Table 1).

**Table 1:** Various N-Benzothiazolyl-2-Phenyl Acetamide derived compounds with substitutions at the six substitution sites, percentage inhibition showed at a concentration of 10 μM and their IC50 values

Compound	R1	R2	R3	R4	R5	R6	IC_50_
1	H	Me	H	Cl	H	H	0.083
2	H	CF_3_	H	Cl	H	H	0.023
3	H	OMe	H	Cl	H	H	0.53
4	H	OCF_3_	H	Cl	H	H	0.54
5	H	OEt	H	Cl	H	H	1.21
6	H	CF_3_	Cl	H	H	H	0.068
7	H	OMe	Cl	H	H	H	9.71
8	H	OEt	Cl	H	H	H	17.43
9	H	CF_3_	H	H	Cl	H	0.065
10	H	OMe	H	H	Cl	H	0.75
11	H	OEt	H	H	Cl	H	1.11
12	H	Br	OMe	H	H	H	0.26
13	H	Cl	OMe	H	H	H	0.32
14	H	F	OMe	H	H	H	1.17
15	H	Me	OMe	H	H	H	0.29
16	H	OMe	OMe	H	H	H	2.22
17	H	OCF_3_	OMe	H	H	H	0.62
18	H	OEt	OMe	H	H	H	5.76
19	H	CF_3_	H	OMe	H	H	0.042
20	H	OMe	H	OMe	H	H	0.42
21	H	OEt	H	OMe	H	H	0.99
22	H	CF_3_	H	CF_3_	H	H	0.087
23	H	CF_3_	H	H	OMe	H	0.033
24	H	OMe	H	H	OMe	H	0.57
25	H	OEt	H	H	OMe	H	1.09
26	H	H	H	H	H	H	0.33
27	H	CF_3_	H	H	H	H	0.047
28	H	CF_3_	H	Cl	Cl	H	0.056
29	H	OMe	H	Cl	Cl	H	1.24
30	H	OEt	H	Cl	Cl	H	3.43
31	H	OCF_3_	H	Cl	Cl	H	0.59
32	H	CF_3_	OMe	H	H	OMe	0.19
33	H	OCF_3_	H	OMe	OMe	OMe	0.079
34	H	OMe	H	OMe	OMe	OMe	1.12
35	H	OEt	H	OMe	OMe	OMe	1.43
36	H	CF_3_	H	OMe	OMe	OMe	0.015
37	H	H	H	Cl	H	H	0.85

### Creation of training set and test set

The dataset that was used during the course of this study consisted of a total of 37 molecules. These molecules were manually divided into training and test set so as to keep a uniform distribution of active as well as inactive molecules in both the sets. The selected 37 compounds were divided into test set (30% of dataset) and training set (70% of dataset) to keep a balance ratio as also studied in different GQSAR studies [25, 35, 42]. Molecules 2, 11, 14, 15, 20, 23, 28, 29 and 33 were taken into the test set whereas the others were included in the training set.

### Building of the GQSAR model

For building the GQSAR model, various Variable Selection and Model Building methods are used and implemented such as Step-wise Forward/Backward/Forward-Backward, Simulated Annealing, Genetic Algorithm methods for Variable Selection and Multiple Regression, Partial Least Square, Principal Component Regression methods for Model Building. In this study, Stepwise Forward variable selection method was employed in order to choose from the pool of descriptors, a subset of descriptors. The Step-wise Forward selection method begins with developing a trial model one step at a time with only one independent variable. At each step, the independent variables are added one by one and the model is refitted accordingly. This process is terminated if the last variable that enters the model has regression coefficient which is insignificant or if all the variables have been included in the model [43].

For model building, the Partial Least Square method was used. This method relates a matrix, say Y, of dependent variables (like biological activities of the molecule) to another matrix, say X, of independent variables (like physico-chemical descriptors). The two principle objectives of this method are to approximate the two matrices and to reduce correlation between them. In this method, matrix X is decomposed into several latent variables which correlate best with the molecule’s activity [44]. Variable Selection and Model Building wizard in VLifeMDS was utilized for this purpose.

### Validation and evaluation of the model

Various statistical parameters such as r^2^, q^2^, predicted r^2^ and F-test were used to analyze goodness of fit of the QSAR model developed. Squared correlation coefficient, r^2^ is the square of the correlation between the response values and the predicted response values. It can take any value between 0 and 1. However, a value closer to 1 indicates that a greater proportion of variance is accounted for by the model. F-test is used for comparing statistical models which have been fitted to a dataset for identifying a model which is best fitted. A high F-test value indicates statistical significance of the model reducing the possibility of failure of the model. Low standard errors, r^2^_se, q^2^_se, pred_r^2^_se hint at lower probability of failure of the model and show that the quality of the model is high [25]. The developed model s considered to be robust it fulfils the following conditions- *r* ^*2*^ > 0.6, q^2^ > 0.6, pred_r^2^ > 0.5 [45–47].

### Cross-validation of the model

The developed QSAR model can be cross validated by using internal and external validation methods. The internal validation of the model was carried out by using the Leave One Out (LOO) method. The leave one out cross validated correlation coefficient, q^2^, is used as a fitting function for the evaluation of the models. This method uses a single observation as the validation data from the sample and the remaining observations are taken as training set. This procedure is repeated in a way that every observation is used at least once as validation data. For calculating q^2^, each compound in the training set is sequentially removed and the model is refitted using the same descriptors; the biological activity of the removed molecule being predicted with the help of the refit model [25].

The formula which calculates q^2^ is-

$$ \mathrm{pred}\_{\mathrm{r}}^2=1-\frac{{\displaystyle \sum}\left({\mathrm{y}}_{\mathrm{i}}-{\widehat{\mathrm{y}}}_{\mathrm{i}}\right){}^2}{{\displaystyle \sum}\left({\mathrm{y}}_{\mathrm{i}}-{\mathrm{y}}_{\mathrm{mean}}\right){}^2} $$

Where,

- ${\mathrm{y}}_{\mathrm{i}}$ = actual activity of the i^th^ molecule in the training set
- ${\hat{\mathrm{y}}}_{\mathrm{i}}$ = predicted activity of the i^th^ molecule in the training set
- ${\mathrm{y}}_{\mathrm{mean}}$ = average activity of all the molecules in the training set

The external validation of the model was carried out by calculating the predicted r^2^. This indicates how well the calculated model predicts responses for new observations. Predicted r^2^ is calculated by removing each molecule/observation from the dataset systematically and then determining how well the removed observation has been predicted by the model. An important benefit of predicted r^2^ is that it helps in the prevention of overfitting the model.

It is calculated by the following formula-

$$ \mathrm{pred}\_{\mathrm{r}}^2=1-\frac{{\displaystyle \sum}\left({\mathrm{y}}_{\mathrm{i}}-{\widehat{\mathrm{y}}}_{\mathrm{i}}\right){}^2}{{\displaystyle \sum}\left({\mathrm{y}}_{\mathrm{i}}-{\mathrm{y}}_{\mathrm{mean}}\right){}^2} $$

Where,

- ${\mathrm{y}}_{\mathrm{i}}$ = actual activity of the i^th^ molecule in the test set
- ${\hat{\mathrm{y}}}_{\mathrm{i}}$ = predicted activity of the i^th^ molecule in the test set
- ${\mathrm{y}}_{\mathrm{mean}}$ = average activity of all the molecules in the test set

### Generation of combinatorial library

A combinatorial library was created using the LeadGrow module of VLifeMDS [40]. This was done by substituting various groups at the six different substitution sites of the N-Benzothiazolyl-2-Phenyl Acetamide template which represents the common substructure of the experimentally reported dataset (Fig. 1). The library thus created was generated by making different permutations and combinations of the substituents at the substitution sites and it was comprised of a total of 10,000 compounds. The GQSAR model was used to predict the activity of the compounds generated in the library.

### Docking of the ligands with CK-1δ protein

The ligands with highest predicted activity were selected for docking studies and were prepared using the LigPrep utility of Schrodinger [48, 49]. With the help of this, energy minimized 3-D structures of the compounds were generated. The protein CK-1δ used in this study was obtained from Protein Data Bank (PDB Id- 3UYS). This protein was prepared for docking using the Protein Preparation Wizard of Glide [50]. CK-1δ protein preparation involved capping of termini, removal of water molecules, treatment of disulfides and addition of explicit hydrogen. This was followed by the optimization of the protein after which a grid was generated around the interacting residues making use of the Receptor Grid Generation utility of Schrodinger [49]. The ligands prepared with the help of LigPrep were docked with the protein, CK-1δ using the GlideXP module which resulted in their binding affinities [51, 52]. The ligands were ranked on the basis of their binding affinities. The one exhibiting the best binding affinity was merged with the protein and the resulting complex was separately analyzed. This step yielded top two compounds with high binding affinities. With the aim of getting better insight into the binding mode, the compound exhibiting the highest activity amongst the N-Benzothiazolyl-2-Phenyl Acetamide derivatives was also docked with CK-1δ protein and its binding affinity was recorded.

## Results and discussions

### Descriptors calculation and validation of the data in training and test set

The VLifeMDS software calculated a total of 1027 descriptors. These descriptors were preprocessed by the removal of invariable columns which resulted in a total of 373 descriptors. Nine compounds (2, 14, 15, 20, 23, 28, 29 and 33) were incorporated in the test set whereas the remaining compounds were included in the training set. The test set was chosen as maximum pIC50 value of the test set compound was less than or equal to that of the training set and the lowest pIC50 value of the test compound was more than or equal to that of the training set. This confirms that the test set has been derived from the maximum-minimum range of the train set and is interpolative. Unicolumn statistics of the training set and the test set were obtained (Table 2).

**Table 2:** Unicolumn statistics of the test and training set data for CK-1δ inhibitory activity

Column Name	Average	Maximum	Minimum	Standard Deviation	Sum
Training Set	6.2470	7.8239	4.7587	0.7148	174.9167
Test Set	6.8667	7.6383	5.9318	0.6214	61.8006

### Analysis of the GQSAR model

Using Stepwise Forward variable selection and Partial Least Square model building method, a robust GQSAR model best explaining the biological activity of the N-Benzothiazolyl-2-Phenyl compounds as a function of certain site-specific physico-chemical descriptors, was obtained. The model can be represented as follows-

$$ \mathrm{pIC}50 = 0.8709\times \left(\mathrm{R}2-\mathrm{slogp}\right)-1.2447\times \left(\mathrm{R}3-\mathrm{P}\mathrm{s}\mathrm{i}1\right)-0.6798\times \left(\mathrm{R}2-\mathrm{SssCH}2\mathrm{Count}\right) + 0.1867\times \left(\mathrm{R}6-\mathrm{HydrogensCount}\right)+5.9327 $$

with *n* = 28, degree of freedom = 25, r^2^ = 0.90, r2_standard error = 0.24, q^2^ = 0.85, q^2^_standard error = 0.29, pred_r^2^ = 0.89, pred_r^2^_standard error = 0.30, F test = 108.59.

Here,

- n = no. of compounds in regression
- r^2^ = correlation coefficient (squared)
- q^2^ = squared correlation coefficient (cross validated)
- Pred_r^2^ = predicted squared correlation coefficient
- F-test = denotes that the results are not just based on chanced correlations

This model satisfies all the statistical parameters such as *r* ^*2*^ > 0.6, q^2^ > 0.6, pred_r^2^ > 0.5. It also exhibits a very high F-test value and very low standard errors supporting the robustness of the model. The GQSAR model also indicates the influence of the four descriptors namely R2_slogp, R3_Psi1, R2_SssCH2Count and R6_HydrogensCount on their respective substitution sites.

**Fig. 2:**
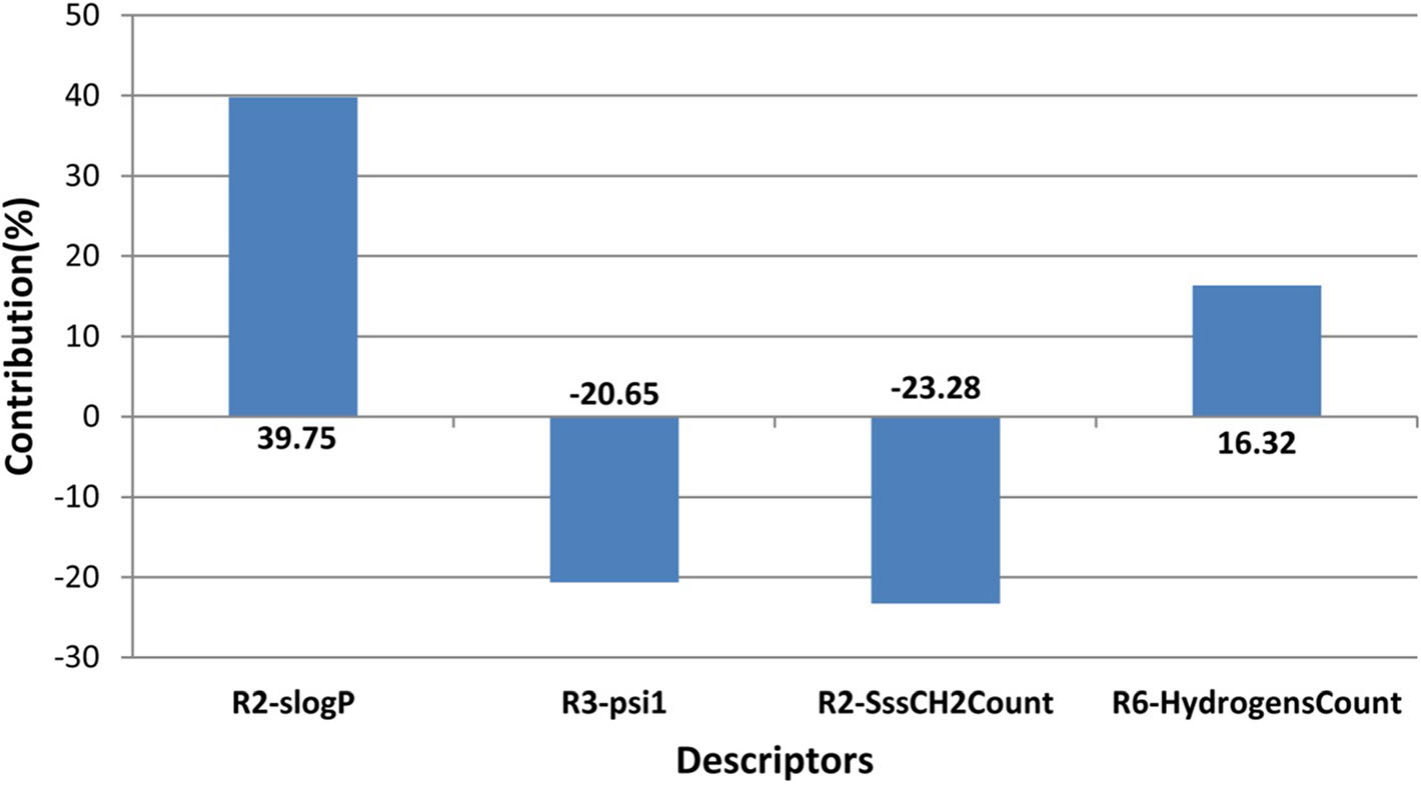
Contribution Plot depicting positive and negative contribution of the four descriptors of developed GQSAR model

The descriptor, R2_slogp, belongs to the sub class Individual. It describes the log of octanol/water partition coefficient and calculates the log *p* value from the structure which is given. This indicates the contribution of the descriptor at the R2 substitution site (Table 3). This descriptor has a positive contribution of 39.75%, as is evident from the contribution plot (Fig. 2) suggesting that the presence of hydrophobic groups at this position would enhance the inhibitory activity of the compound. The second descriptor, R3_Psi1, is a member of the sub class Extended Topochemical Atom Based Descriptors which gives a measure of the tendency of the molecules for hydrogen bonding or the polar surface area of molecules. It exhibits a negative contribution of 20.65% at the R3 substitution site indicating that an increase in the polar surface area of the molecule or the number of molecules capable of forming hydrogen bond may decrease the inhibitory action of the compound. The third descriptor, R2_SssCH2Count, belongs to the sub class Estate Numbers. It gives an indication about the total number of –CH2 groups which are connected with the help of two single bonds. It is shown to have a negative contribution of 23.28% at R2 substitution site of the compound hinting that a reduction in such groups would be better for the inhibitory activity of the compound. The final descriptor, R6_HydrogensCount, belongs to the sub class Element Count which is an indicator of the number of Hydrogens present in a particular compound. At R6 substitution site, this descriptor effects a positive contribution of 16.32% indicating the importance of hydrogen atoms at this site for a better inhibitory activity.

**Table 3:** Contribution of various physico-chemical descriptors

Compound No.	pIC_50_ Value	R2-slogp	R2-SssCH2Count	R6-HydrogensCount	R3-Psi1
1	7.0809	0.6361	0	2	Nan
2	7.6382	1.1785	0	2	Nan
3	6.2757	−0.3915	0	2	Nan
4	6.2676	0.4985	0	1	Nan
5	5.9172	0	0	0	0
6	7.1674	1.1785	0	2	0.5154
7	5.0127	−0.3915	0	2	0.5154
8	4.7587	−0.0014	1	2	0.5154
9	7.1870	1.1785	0	2	Nan
10	6.1249	−0.3915	0	2	Nan
11	5.9546	−0.0014	1	2	Nan
12	6.5850	0.9581	0	2	0.3846
13	6.4948	0.802	0	2	0.3846
14	5.9318	0.5327	0	2	0.3846
15	6.5376	0.6361	0	2	0.3846
16	5.6536	−0.3915	0	2	0.3846
17	6.2076	0.4985	0	2	0.3846
18	5.2395	−0.0014	1	2	0.3846
19	7.3767	1.1785	0	4	Nan
20	6.3767	−0.3915	0	4	Nan
21	6.004	−0.0014	1	4	Nan
22	7.0604	1.1785	0	1	Nan
23	7.4814	1.1785	0	2	Nan
24	6.2441	−0.3915	0	2	Nan
25	5.9625	−0.0014	1	2	Nan
26	6.4814	0.246	0	2	Nan
27	7.3279	1.1785	0	2	Nan
28	7.2518	1.1785	0	1	Nan
29	5.9065	−0.3915	0	1	1.7976
30	5.4647	−0.0014	1	1	Nan
31	6.2291	0.4985	0	1	Nan
32	6.7212	1.1785	0	4	0.3846
33	7.1023	0.4985	0	4	Nan
34	5.9507	−0.3915	0	4	Nan
35	5.8446	−0.0014	1	4	Nan
36	7.8239	1.1785	0	4	Nan
37	6.0705	0.246	0	2	Nan

**Fig. 3:**
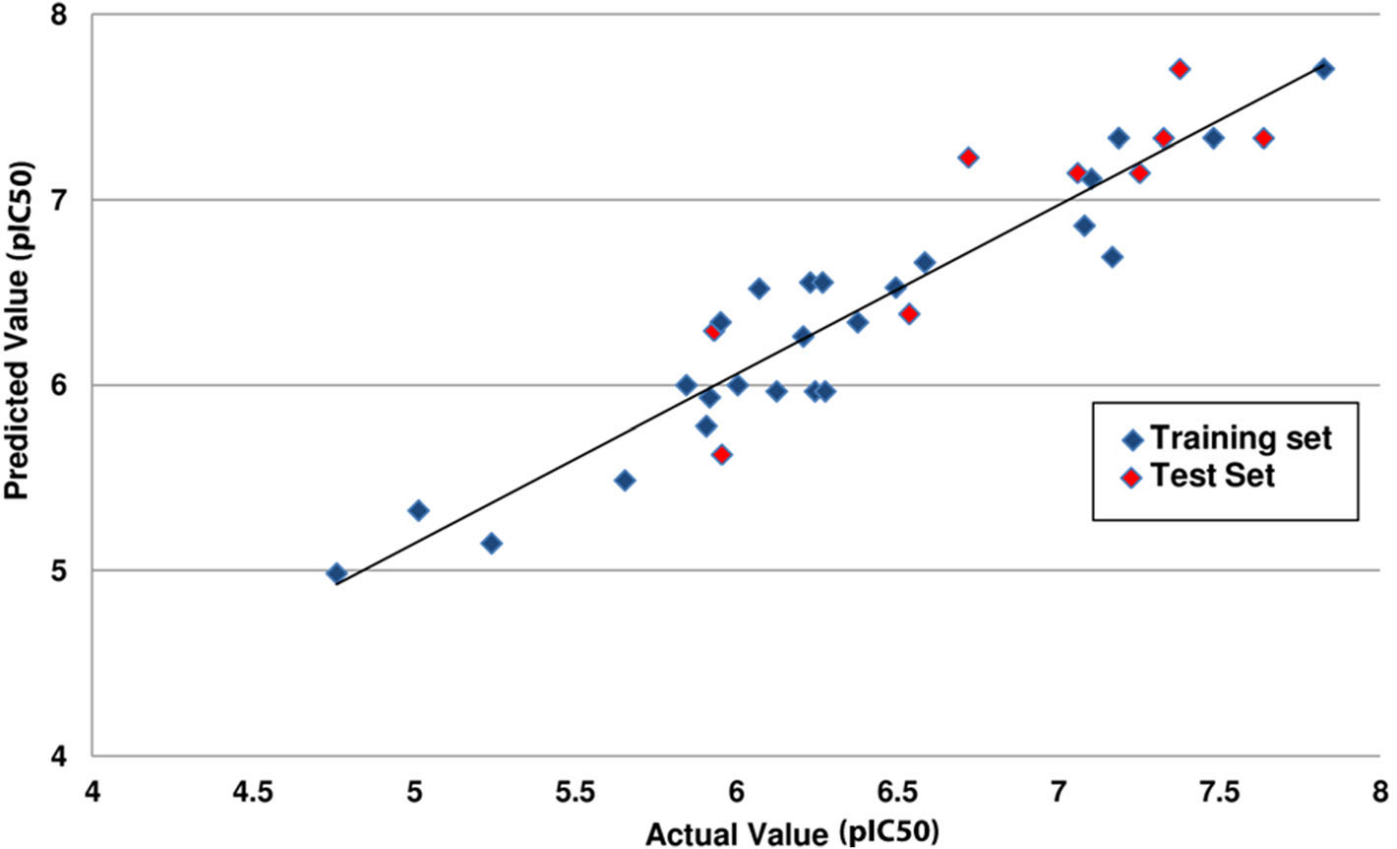
Graph of observed/actual vs. predicted activity of the test and training set data

**Fig. 4:**
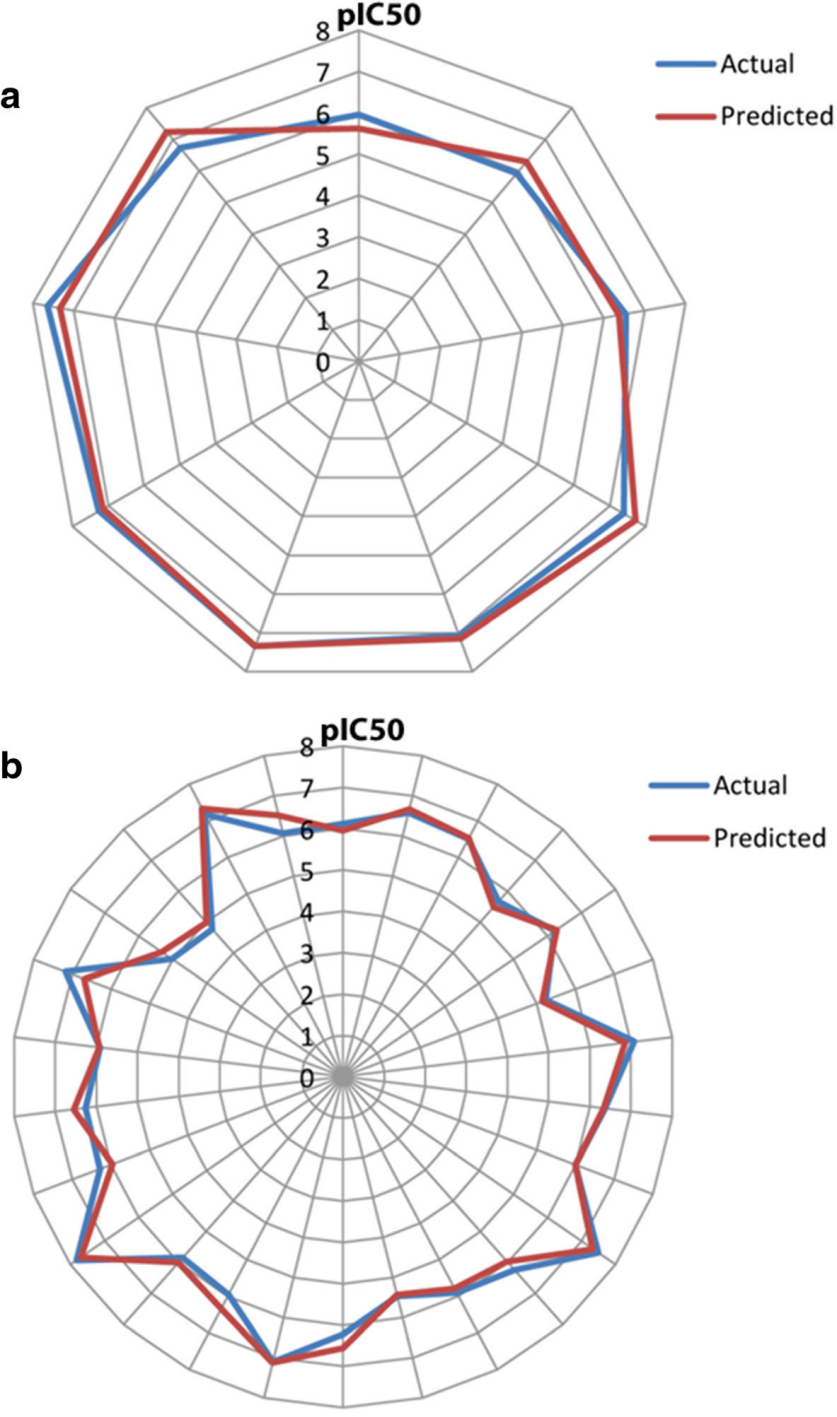
Radar Plots representing predicted and observed/actual activity values of (**a**) test set and (**b**) training set

Minimal difference between the actual and predicted values of the compounds is a measure of high quality of the model [53, 54]. The significance of a model is described by its various statistical parameters. A high value of the squared correlation coefficient, 0.90, along with very low standard error, 0.23, indicates that the model is highly accurate. Good internal predictive power of the model can be judged by a very high value of cross validated correlation coefficient. Similarly, the value of predicted squared correlation coefficient, 0.89, indicates that the model has good external predictive ability. Since a high F-test value was obtained, 108.59, it can be assured that there are very few chances that the model will fail. Low standard error values represent the fact that the quality of the model generated is very high and the model is predictive and reliable. A Fitness Plot (Fig. 3) and Radar Plots (Fig. 4) were obtained which represent and compare the actual and predicted activities of the molecules of the training set and the test set.

### Analysis of the combinatorial library generated using the GQSAR model

The combinatorial library was created by substituting the various sites with rings, aromatic rings, alkyl groups and atoms. The inhibitory activities of the compounds generated were predicted using the GQSAR model generated previously. Around 10,000 compounds were generated in the combinatorial library whose predicted activities ranged from 3.83 to 39.44. Out of these 10,000 compounds, 240 compounds had predicted activity more than the highest activity of the compounds of the experimentally reported dataset (pIC50 = 7.8). The substituents in these compounds exhibiting high predictive power were rings at R1 and R4 positions, alkyl groups at R2 position, electronegative atoms such as fluorine and oxygen at R3 position, carbonic acids and acetate esters at R5 and aromatic rings at R6 position. The presence of highly electronegative atoms at R3 plays the most important role in deciding the activities of compounds. The compounds with atoms other than fluorine and oxygen display lower activity values as compared to those with these two atoms.

### Docking analysis of the designed lead compounds with CK-1δ

The compounds which exhibited the best predicted inhibitory values, more than the highest value of the experimentally reported dataset, were selected for further docking analysis. Top two compounds were reported as potent lead compounds against CK-1δ.

**Fig. 5:**
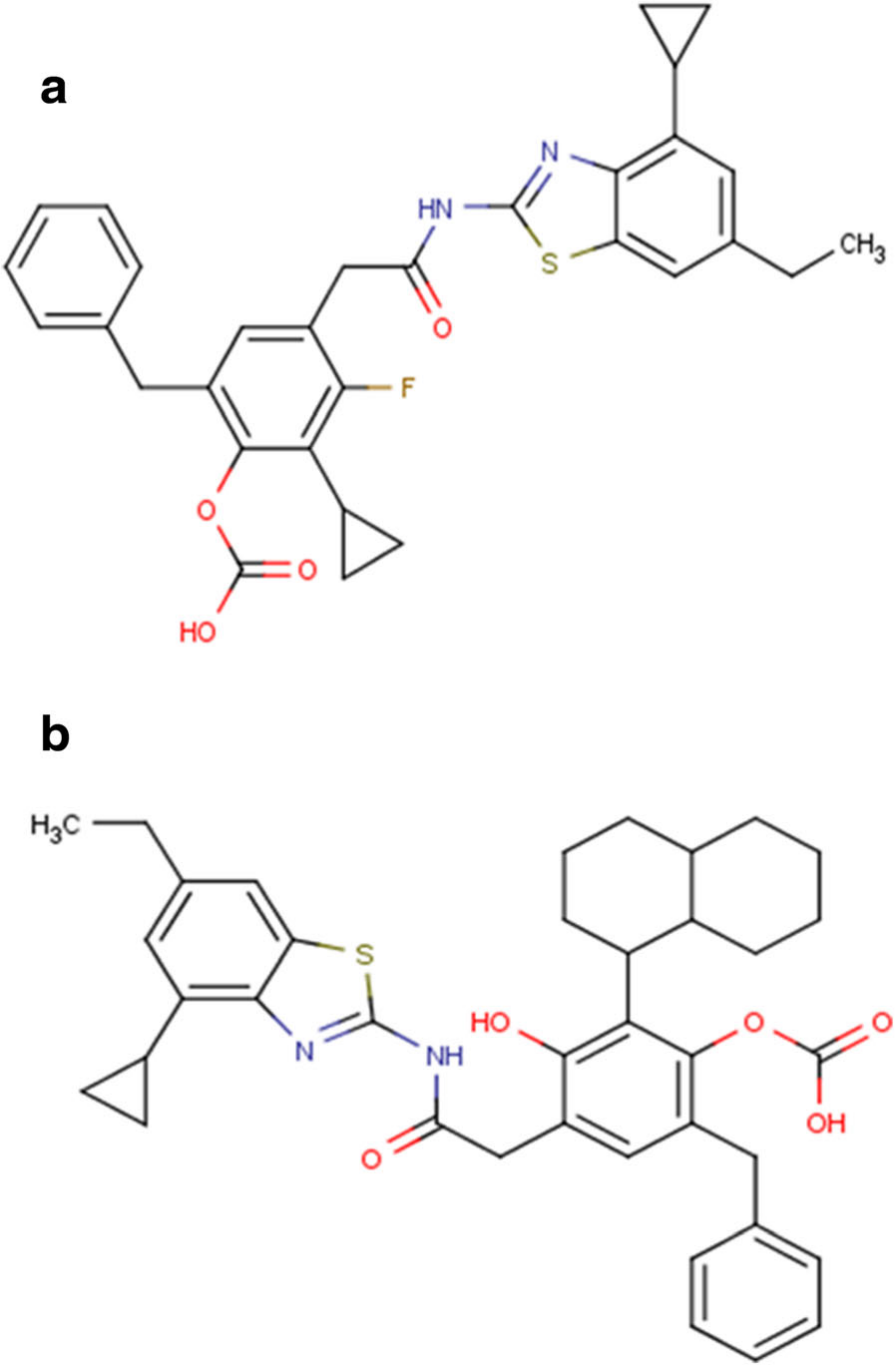
Structures of the two highly active compounds (**a**) CHC (6-benzyl-2-cyclopropyl-4-{[(4-cyclopropyl-6-ethyl-1,3-benzothiazol-2-yl)carbamoyl]methyl}j-3-fluorophenyl hydrogen carbonate) and (**b**) DHC (6-benzyl-4-{[(4-cyclopropyl-6-ethyl-1,3-benzothiazol-2-yl)carbamoyl]methyl}-2-(decahydronaphthalen-1-yl)-3-hydroxyphenyl hydrogen carbonate). (Heteroatoms are shown in different colors; as Nitrogen in *blue*, oxygen in *red* and sulfur in *green*)

The first compound, 6-benzyl-2-cyclopropyl-4-{[(4-cyclopropyl-6-ethyl-1,3-benzothiazol-2-yl)carbamoyl]methyl}j-3-fluorophenyl hydrogen carbonate (CHC) (Fig. 5a) consisted cyclopropane at R1, ethyl at R2, fluorine at R3, another cyclopropane at R4, carbonic at R5 and a benzyl group at R6. This compound displayed a binding score of −6.11 Kcal/mol. The other compound, 6-benzyl-4-{[(4-cyclopropyl-6-ethyl-1,3-benzothiazol-2-yl)carbamoyl]methyl}-2-(decahydronaphthalen-1-yl)-3-hydroxyphenyl hydrogen carbonate (DHC) (Fig. 5b) had cyclopropane at R1, ethyl at R2, hydroxyl at R3, cyclobutane at R4, carbonic at R5 and naphthalene moiety at R6. This compound possessed a binding score of −6.01 Kcal/mol. The various components of the Glide score of these two compounds are provided in Table 4. The compound N-[6-(trifluoromethyl)-1,3-benzothiazol-2-yl]-2-(3,4,5-trimethoxyphenyl)Acetamide (BTA), which exhibited the highest inhibitory activity in the experimental dataset with a pIC50 value of 7.8, was also docked with CK-1δ for a comparative analysis. The GlideXP score of this particular compound was −3.78 Kcal/mol, suggesting that the compounds designed (CHC and DHC) had better binding affinities for CK-1δ protein than the experimentally reported compounds. Structure analysis of the novel leads make it clear that both the compounds have cyclopropane ring at R1, ethyl at R2 and carbonic group at R5 in common. CHC is more active displaying higher binding score, having another cyclopropane ring at R4, a fluorine at R3 and another single six membered (benzene) ring at R6 in comparison to DHC that contain butane ring, hydroxyl group and a fused pair of six membered rings (naphthalene) at the respective positions.

**Table 4:** Glide score XP and its components

Complex	CHC-CK-1δ	DHC-CK-1δ	BTA-CK-1δ
Glide Score XP	−6.11	−6.01	−3.78
Glide Hydrogen Bond	−0.13	−0.36	−0.20
Glide Evdw	−36.68	−42.51	−35.93
Glide Ecoul	−17.69	−13.02	−5.10
Glide Emodel	−61.95	−79.92	−38.17
Glide Energy	−52.37	−55.53	−41.03

### Interactions of CHC, BHC with CK-1δ protein

Docking analysis of the top scoring compounds provided insights into the mode of interactions of the designed compounds with the protein Casein Kinase-1δ. Molecular binding is a phenomenon that relies on the entropy-enthalpy compensation and contain both entropic and enthalpy components. The binding may be entropy driven in case of the hydrophobic effect or enthalpy driven in case of the dominant non-covalent attractive forces. Fundamentally, both the entropic and enthalpy component must result into a negative Gibbs’ free energy for effective binding. In our study the reported lead molecules show high negative binding free energy in comparison to the in-vitro reported compound which displays a significant binding capability of the lead molecules. The hydrophobic interactions are the most important forces in stabilizing biological structures ranging from native conformations of proteins to cellular membranes. In our study, high negative value of van der Waal energy represents the massive hydrophobic interaction (Table 4) and hydrogen bonds as the non-covalent attractive forces.

**Fig. 6:**
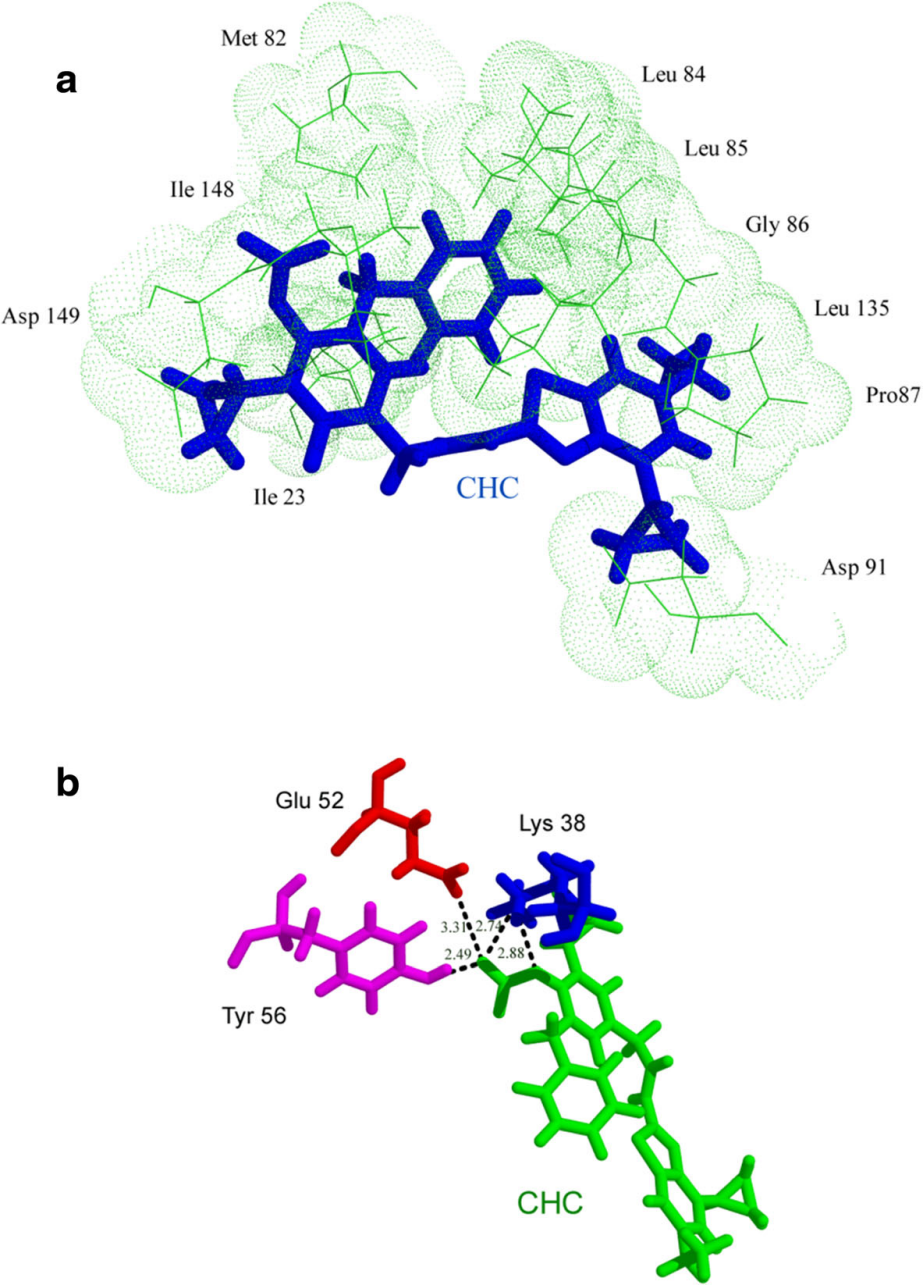
Molecular interactions of CK-1δ with CHC; different colors are used for distinct visualization of interaction and do not relate to nature of molecules or functional difference (**a**) representation of hydrophobic interactions (CHC in *blue* and CK-1δ protein in *green*) and (**b**) hydrogen bonds (CHC in *green* and CK-1δ residues Lys, Glu and Tyr in *blue*, *red* and magenta, respectively

The first compound, CHC displayed four hydrogen bond interactions with three residues of CK-1δ- Glu52, Tyr56 and Lys38. The bond with a length of 3.31 Å was formed with Glutamic acid. The second hydrogen bond of bond length 2.49 Å was formed between the same atom of CHC with Tyrosine. Third and the fourth hydrogen bonds were formed between the fourth oxygen of carbonic group of CHC and Lysine (bond length = 2.74 Å) and the second oxygen atom of carbonic group of CHC and Lysine (bond length = 2.88 Å). CHC also exhibited hydrophobic interactions with several residues such as Asp149, Asp91, Leu135, Ile148, Gly86, Leu84, Leu85, Pro87, Ile23 and Met82 (Fig. 6).

**Fig. 7:**
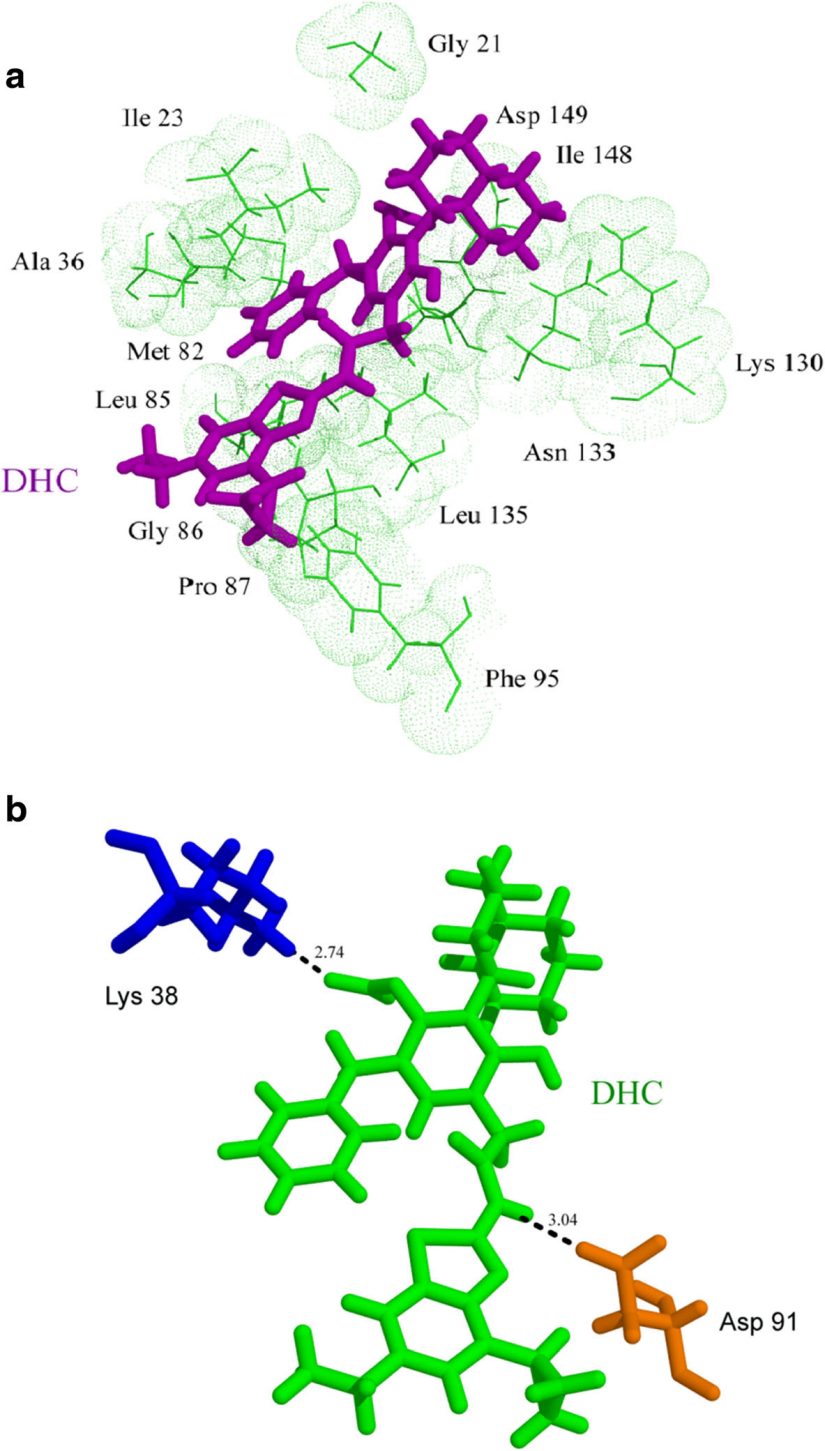
Molecular interactions of CK-1δ with DHC; different colors are used for distinct visualization of interaction and do not relate to nature of molecules or functional difference (**a**) representation of hydrophobic interactions (DHC in *purple* and CK-1δ protein in *green*) and (**b**) hydrogen bonds (DHC in *green* and CK-1δ residues Lys in *blue* and Asp in *yellow*)

The second lead compound DHC exhibited two hydrogen bonds with CK-1δ. The first one was formed between the nitrogen of DHC and Asp91 (bond length = 3.04 Å). The second hydrogen bond was formed between the fifth oxygen of DHC and Lys38 (bond length = 2.74 Å). DHC also exhibited hydrophobic interactions with various residues like Phe95, Lys130, Asn133, Gly21, Ile148, Asp149, Ile23, Met82, Leu85, Leu135, Pro87, Gly86 and Ala36 (Fig. 7). A summary of these interactions is provided in Table 5.

**Table 5:** Various CK-1δ residues involved in different kinds of interactions with CHC and DHC

Complex	Residues involved in hydrophobic interactions	Residues involved in hydrogen bonding
CK-1δ-CHC	Ile23, Met82, Leu84, Leu85, Gly86, Pro87, Asp91, Leu135, Ile148, Asp149	Lys38, Glu52, Tyr56
CK-1δ-DHC	Gly21, Ile23, Ala36, Met82, Leu85, Gly86, Pro87, Phe95, Lys130, Asn133, Leu135, Ile148, Asp149	Lys38, Asp91

The interacting residues in case of both the lead molecules lie in common to the reported ATP binding site residues of the CK-1δ protein. This confirms the structural reasons for inhibitory activity of the lead molecules [1].

## Conclusions

In this study, an attempt was made at creating a novel GQSAR model for the derivatives of N-Benzothiazolyl-2-Phenyl Acetamide which act as inhibitors of Casein Kinase-1δ protein. This protein causes the phosphorylation of TAR DNA Binding Protein-43 (TDP-43), a phenomenon which is associated with the onset and progression of a neurodegenerative disorder, Amyotrophic Lateral Sclerosis (ALS). A QSAR equation was obtained which constituted four descriptors namely, R2-slogp, R3-Psi1, R2-SssCH2count and R6-HydrogensCount. The first descriptor displayed a positive contribution at the substitution site R2 whereas the second one displayed negative contribution at R3. The third descriptor exhibited a negative contribution at R2 and the last descriptor was shown to contribute positively to R6 substitution site. GQSAR model was analysed on various statistical parameters and found to be robust. Internal validation of the model was carried out by the leave one out method and external validation was carried out by predicting the activity of the test set molecules.

A combinatorial library was prepared and the activities of the compounds were predicted using the developed QSAR model. An analysis of the compounds generated from this library revealed that the presence of cyclic rings at R1 and R4, alkyl groups at R2, electronegative atoms such as fluorine and oxygen at R3 acetate esters at R4 and aromatic rings at R6 were beneficial in enhancing the inhibitory activity of the compounds. This was followed by docking, resulting in the top scoring compounds, CHC and DHC, with highest binding affinities with the protein CK-1δ. This study provides substantial amount of evidence that these compounds can be considered as potential leads against the CK-1δ protein, inhibiting the phosphorylation of TDP-43 and thus preventing ALS. These molecules have been developed on the basis of a highly accurate and validated GQSAR model and have also proved to have high binding affinity towards CK-1δ as displayed through the docking analysis. CHC and DHC can be the good leads for further in-vitro testing as CK-1δ inhibitors and have the potential to be include in the drug development pipeline as CK-1δ antagonists.
